# Nutrient intakes and iron and vitamin D status differ depending on main milk consumed by UK children aged 12–18 months – secondary analysis from the Diet and Nutrition Survey of Infants and Young Children

**DOI:** 10.1017/jns.2016.24

**Published:** 2016-07-29

**Authors:** Anne Sidnell, Sandrine Pigat, Sigrid Gibson, Rosalyn O'Connor, Aileen Connolly, Sylwia Sterecka, Alison M. Stephen

**Affiliations:** 1 Nestlé Nutrition, 1 City Place, Gatwick, Crawley RH6 0PA, UK; 2 Creme Global, 4th Floor, The Tower, Trinity Technology and Enterprise Campus, Grand Canal Quay, Dublin 2, Republic of Ireland; 3 Sig-Nurture Ltd, 11 Woodway, Merrow, Guildford, Surrey GU1 2TF, UK; 4 Department of Nutritional Sciences, Faculty of Health and Medical Sciences, University of Surrey, Guildford, Surrey GU2 7JP, UK

**Keywords:** Iron, Vitamin D, Fortified milk, Cows’ milk, Young children, Dietary surveys, 25(OH)D, 25-hydroxyvitamin D, DNSIYC, Diet and Nutrition Survey of Infants and Young Children, EAR, estimated average requirement, RNI, reference nutrient intake

## Abstract

Nutrition in the second year is important as this is a period of rapid growth and development. Milk is a major food for young children and this analysis evaluated the impact of the type of milk consumed on nutrient intakes and nutritional status. Data from the Diet and Nutrition Survey of Infants and Young Children were used to investigate the intakes of key nutrients, and Fe and vitamin D status, of children aged 12–18 months, not breastfed, and consuming >400 g/d fortified milk (*n* 139) or >400 g/d of whole cows’ milk (*n* 404). Blood samples from eligible children for measurement of Hb (*n* 113), serum ferritin and plasma 25-hydroxyvitamin D (25(OH)D) concentrations (*n* 105) were available for approximately 20 % of children. Unpaired Mann–Whitney tests were used to compare nutrient intakes and status between consumers of fortified and cows’ milk. Mean daily total dietary intakes of Fe, Zn, vitamin A and vitamin D were significantly higher in the fortified milk group. Mean daily total dietary intakes of energy, protein, Ca, iodine, Na and saturated fat were significantly higher in the cows’ milk group. Hb was not different between groups. The fortified milk group had significantly higher serum ferritin (*P* = 0·049) and plasma 25(OH)D (*P* = 0·014). This analysis demonstrates significantly different nutrient intakes and status between infants consuming >400 g/d fortified milk *v*. those consuming >400 g/d whole cows’ milk. These results indicate that fortified milks can play a significant role in improving the quality of young children's diets in their second year of life.

The first 1000 d of life, from conception to 2 years of age, are a critical period for growth and development^(^^1^^)^. In the second year of life (12–24 months), young children are undergoing rapid growth and development at the same time as making the transition from weaning foods towards a family diet. This may make them vulnerable to nutrient inadequacy if their diets are restricted or lacking in nutrient-dense foods. In addition their diets may provide excesses of some nutrients or supply more energy than required.

Whole cows’ milk may be introduced as a drink beyond 12 months of age and replacement of a formula (fortified) milk with whole cows’ milk is one of the common dietary changes during this period^(^^2^^)^. Fortified milks (also referred to as growing-up milks), contain less protein, Ca and saturated fat than whole cows’ milk, as well as being fortified with micronutrients including Fe and vitamin D. As milk continues to be an important part of the diet in early childhood, the main milk consumed may have a significant impact on nutritional intakes for this age group.

In 2011, the Diet and Nutrition Survey of Infants and Young Children (DNSIYC) surveyed 2683 UK infants and young children aged between 4 and 18 months^(^^2^^)^. This large study revealed detailed dietary intake data and collected and analysed blood samples for Fe and vitamin D status measures, making this a valuable and unique dataset. Results from the DNSIYC suggest that diet quality changes after 12 months, with a decline in consumption of micronutrient-fortified foods and milks, and an increase in the consumption of foods high in energy and in fat, sugar and salt^(^^2^^)^. Similarly the 2008 Feeding Infants and Toddlers Study in the USA reported increased consumption of nutrient-poor snacks, sweets and soft drinks after 12 months^(^^3^^)^. A deterioration in dietary quality at this age may have implications for nutritional adequacy and risk of overweight, obesity and non-communicable disease in later life.

Across Europe young children's diets have been identified as potentially low in Fe, vitamin D and long-chain PUFA^(^^4^^)^. A recent review of micronutrient intakes for eight member states highlighted low intakes for specific age groups and countries. In terms of Fe, for children from 1 to 3 years, the proportion below the lower reference nutrient intake (RNI) ranged between 3·1 % (Belgium) and 27 % (Poland). Proportions below the estimated average requirement (EAR) were considerable, ranging from 23 % (Belgium) to 55 % (Poland)^(^^5^^)^. In the UK (DNSIYC), 13 % of children aged 12–18 months had Fe intakes below the lower RNI^(^^2^^)^. Children of South Asian ethnicity were significantly more likely to have intakes below the lower RNI compared with white children (28 *v*. 11 %). Status measures showed that 11 % of children aged 12–18 months had serum ferritin levels below 10 µg/l, indicative of low Fe stores, and 15 % had Hb levels below lower reference limits (110 g/l)^(^^2^^)^. The prevalence of Fe-deficiency anaemia (the combination of low serum ferritin and low Hb) was 2 % in this age group. These values for Fe status were similar to those from the Avon Longitudinal Study of Parents and Children^(^^6^^)^.

Dietary intake of vitamin D is of universal concern across Europe due to low intakes. In the DNSIYC, dietary vitamin D intake, including supplements, was substantially lower in those over 12 months, compared with those aged 10–11 months, averaging only 55 % of the RNI, compared with 111 % (non-breastfed, and including supplements). Of those aged 12–18 months, 31 % had plasma 25-hydroxyvitamin D (25(OH)D) < 50 nmol/l, with 2 % reaching the criterion of vitamin D deficiency, <25 nmol/l^(^^2^^)^.

There is evidence that both energy and protein intakes may be higher than necessary, and that high-protein diets in infancy and early childhood encourage rapid weight gain and increased risk of overweight and obesity in later life^(^^7^^)^. In a multicentre European randomised controlled trial, infant formula with a lower protein content consumed throughout the first year of life was associated with lower weight-for-age at 2 years^(^^8^^)^ than high-protein formula; later follow-up confirmed a lower BMI at 6 years of age^(^^9^^)^. In a German prospective study (DOrtmund Nutritional and Anthropometric Longitudinally Designed (DONALD)), a consistently high protein intake at 12 months and 18–24 months was positively associated with BMI and body fat mass at 7 years of age^(^^10^^)^. Dairy protein intake at 12 months (adjusted for energy intake) was significantly associated with increased percentage body fat and BMI standard deviation score, while protein intake from meat or cereal showed no association^(^^10^^)^. A recent review to inform revision of the Nordic Nutrition Recommendations suggested a mean intake of 15 % energy from protein as an upper limit at 12 months^(^^11^^)^. In the DNSIYC, mean protein intakes were 15·6 % of energy at 12–18 months and were 15·4 % of energy at 1·5–3 years in the National Diet and Nutrition Survey 2008–2012^(^^12^^)^.

## Methods

### Data used in analysis (Diet and Nutrition Survey of Infants and Young Children)

The DNSIYC^(^^2^^)^ is a nationally representative survey conducted in all four countries of the UK, providing data on 2683 infants and young children aged between 4 and 18 months of age. The survey consisted of detailed face-to-face interviews to gather information on dietary habits, feeding practices, sociodemographic status and health information. Dietary intake data were recorded using an estimated (unweighed) food diary on four consecutive days, where parents and/or carers recorded the type and amount of each food and drink consumed by the participant at each eating event, including any dietary supplements given. A detailed explanation of the exact methodology adopted for the DNSIYC is given elsewhere^(^^2^^)^. Except in a boosted sample in Scotland, all those who completed at least three complete days of recording were invited to a second stage of the survey where blood samples were taken from those giving consent, as well as recording of further anthropometric measurements. Blood samples were analysed for Hb, ferritin and 25(OH)D to assess Fe and vitamin D status. For the entire DNSIYC cohort, full blood count was successfully measured in 22 % of the participants; 25(OH)D in 21 %; and serum ferritin in 22 % of participants. The parents of children who gave blood tended to be older than parents of the same aged children in the UK population who did not give blood. This was probably because of lower rates of clinic attendance amongst younger mothers. There were also some regional variations due to differences in response and clinic attendance. However, the demographic profiles of those who went to clinic and the entire DNSIYC UK population were generally similar and there was no evidence of large biases^(^^2^^)^.

### Subjects

In the present analysis, dietary data for non-breastfed children aged 12–18 months (*n* 1174) were used. From these subjects, two subpopulations comprising the following consumer types were extracted: (1) fortified milk consumers who consumed >400 g/d of fortified milk (including follow-on milk and toddler milk/growing up milk) (*n* 139); and (2) whole cows’ milk consumers who consumed >400 g/d of whole cows’ milk (*n* 404). The DNSIYC survey recorded intake of various kinds of milk, and in order to identify participants having whole cows’ milk, the data were regrouped. The remainder who were breastfed (*n* 100), or who consumed other milks, or <400 g fortified or whole cows’ milk (*n* 631) were excluded from the analysis. For this analysis the milk intake cut-off of 400 g/d was therefore selected as a realistic reflection of average milk intake, and to enable an observable difference in the diets of the two groups. This volume of milk equates to 400 ml, 0·7 pints, 11·4 fluid ounces (UK), or 13·5 fluid ounces (USA).

Total daily dietary intakes of energy and nutrients (including contribution from supplements) were compared between the two groups. Nutrients assessed were Ca, energy, iodine, Fe, non-milk extrinsic sugars, protein, SFA, Na, vitamin A, vitamin D and Zn. These were chosen based on the findings of the level of dietary imbalance in young children's diets^(^^12^^)^ or because there are targets and recommendations for dietary intakes of these nutrients. The impact of fortified milks on long-chain PUFA intakes was not examined because the DNSIYC dietary database does not identify these fatty acids, only ‘all *cis n*-3 PUFA’ which would include α-linolenic acid. In addition to total dietary intakes, the mean daily energy and nutrient contribution from the milk consumed (fortified or whole cows’ milk) was obtained, and is presented as an absolute amount and a percentage of the mean total daily intake. Nutrient intakes were compared with RNI and EAR cut-offs to assess the likelihood of dietary inadequacies in both consumer groups (Table 1). All EAR and RNI values are based on the UK Department of Health (1991)^(^^13^^)^, with additional values obtained for energy from the Scientific Advisory Committee on Nutrition (SACN)^(^^14^^)^.

**Table 1.** Reference nutrient intake (RNI) and estimated average requirement (EAR) values used in assessing nutrient intakes

Nutrient	Age (years)	RNI/EAR
Fe (EAR)	1–3	5·3 mg/d
Vitamin D (EAR)	1–3	5·25 µg/d*
Vitamin A (EAR)	1–3	300 µg retinol equivalents/d
Zn (EAR)	1–3	3·8 mg/d
Ca (EAR)	1–3	275 mg/d
Energy (EAR)	1	3·2 MJ/d (boys)
		2·9 MJ/d (girls)
		Average: 3·1 MJ/d or 741 kcal/d†
Protein (RNI)	1–3	14·5 g/d
Na (RNI)	1–3	775·2 mg/d (based on a maximum level of 2 g/d salt)
Non-milk extrinsic sugars (RNI)	1–3	11 % of food energy
Iodine (EAR)	1–3	52·5 µg/d*
SFA (RNI)	1–3	11 % of food energy

* 75 % of RNI (this value was applied as EAR values were not available).

† Average EAR values for energy were calculated for boys and girls combined for the present analysis, as EAR values are calculated based on sex.

Fe status of consumers of fortified milk and cows’ milk was assessed using serum ferritin and Hb concentrations, while vitamin D status was assessed using 25(OH)D plasma concentrations. The threshold values and numbers of participants in each group who provided blood samples for analysis are shown in Table 2^(^^15^^–^^17^^)^.

**Table 2.** Iron and vitamin D threshold values, and numbers and percentages of participants providing blood samples for analysis

		Fortified milk consumers		Cows’ milk consumers	
Blood analyte measure	Threshold value	*n*	%	*n*	%
Hb (for children aged 10 months or over)	110 g/l	35	25	78	19
Serum ferritin (for children aged 12 months and over)	12 µg/l	30	22	75	19
Plasma 25(OH)D (for entire population)	25 nmol/l	30	22	75	19

25(OH)D, 25-hydroxyvitamin D.

### Analysis and statistical comparison of consumer types

Nutrient intakes, including supplements, and status were compared between consumers types using the Creme Nutrition® model^(^^18^^,^^19^^)^, which is scientific, cloud-based software used to assess dietary intakes of foods, chemicals and nutrients in populations of consumers. Statistical tests were carried out to compare nutrient intakes, and vitamin D and Fe status between consumers of fortified milk and consumers of whole cows’ milk. For all statistical tests the unpaired Mann–Whitney test was applied^(^^19^^)^.

## Results

Of the entire DNSIYC 12–18 months group, not breastfed (*n* 1174), 93 % (*n* 1090) consumed milk. Whole cows’ milk was the predominantly consumed milk, and some children consumed both whole cows’ milk and fortified milk as shown in Table 3. Milk intakes of the subgroups consuming more than 400 g/d fortified or whole cows’ milk are shown in Table 4.

**Table 3.** Daily milk consumption (g/d) in Diet and Nutrition Survey of Infants and Young Children population of children aged 12–18 months (milk consumers, non-breastfed)

		Milk intake (g/d)		
	*n*	P2·5	Median	P97·5
Whole cows’ milk	959	14	357	786
Fortified milk	380	43	350	775
All milks combined	1090	47	431	828

P, percentile.

**Table 4.** Daily milk consumption (g/d) in subgroups consuming more than 400 g/d (non-breastfed)

		Milk intake (g/d)		
	*n*	P2·5	Median	P97·5
Fortified milk consumers	139	406	509	895
Whole cows’ milk consumers	404	404	516	849

P, percentile.

The nutrient contributions of fortified milks and whole cows’ milk to total dietary intakes are presented in Table 5 for the two consumer groups. Fortified milk consumers had significantly higher mean daily intakes of Fe, Zn and vitamins A and D than the cows’ milk consumers. Cows’ milk consumers had significantly higher mean daily intakes of energy, protein and Ca compared with fortified milk consumers. There was no difference in the intake of non-milk extrinsic sugars between the groups. Consumers of whole cows’ milk had significantly higher protein intakes than those consuming fortified milks. Fortified milks contributed 25 % of total protein intake in the group consuming fortified milk while cows’ milk contributed 41 % of total protein intake in the cows’ milk consumer group.

**Table 5.** Contribution of milks (fortified and whole cows’ milk) to total intakes of energy, protein, SFA, non-milk extrinsic sugars, vitamin D, vitamin A, iron, zinc, calcium, iodine and sodium in fortified milk consumers (*n* 139) and whole cows’ milk consumers (*n* 404) including *P* value comparing both consumer intake distributions

	Fortified milks							Whole cows’ milk							
Nutrient	Mean daily intake from total diet	Median intake from total diet	IQR	Mean daily intake from fortified milk	% Contribution from fortified milk	Median daily intake from fortified milk	IQR	Mean daily intake from total diet	Median intake from total diet	IQR	Mean daily intake from whole cows’ milk	% Contribution from whole cows’ milk	Median daily intake from whole cows’ milk	IQR	*P*
Energy (kcal)	983	981	842, 1104	376	38	340	309, 413	1046	1029	924, 1148	367	35	343	303, 406	<0·01
Protein (g)	33·8	32·9	28·7, 39·6	8·5	25	7·8	6·9, 9·7	44·1	43·3	38·6, 49	18·2	41	17	15·2, 20·4	<0·01
SFA (g)	14·8	21·9	19, 25	6·3	43	12·5	11, 14·9	22·2	14·4	11·8, 17·9	13·3	60	6·2	4·1, 7·6	<0·01
Non-milk extrinsic sugars (g)	19·8	16·4	10·3, 22·5	3·2	16	0	0, 0	18·7	16·3	11·8, 23·2	0·0	0	0	0, 0	0·64
Vitamin D (μg)	10·5	9·6	8·5, 11·9	8·5	81	8	6·9, 9·2	1·9	1·2	0·8, 2	0·0	0	0	0, 0	<0·01
Vitamin A (μg)	992	929	682, 1238	373	38	341	305, 407	669	594	428, 809	166	25	157	134, 186	<0·01
Fe (mg)	10·4	10·3	8·9, 11·4	6·4	61	5·9	5·2, 7·1	5·1	5	3·9, 6·2	0·0	0	0	0, 0	<0·01
Zn (mg)	7·3	7	6·2, 8·2	4·3	59	4	3·7, 4·8	5·4	5·3	4·7, 6·1	2·2	41	2·1	1·8, 2·5	<0·01
Ca (mg)	795	750	648, 918	417	52	382	333, 475	996	978	849, 1118	644	65	603	532, 720	<0·011
Iodine (μg)	134	127	106, 149	67	50	61	55, 73	255	250	217, 286	204	80	194	169, 233	<0·01
Na (g)	0·75	0·71	0·53, 0·92	0·14	18	0·13	0·11, 0·16	0·99	0·96	0·79, 1·16	0·23	23	0·21	0·19, 0·26	<0·01

IQR, interquartile range.

Among fortified milk consumers, fortified milk was the primary source of vitamin D, with 8·5 µg/d (81 %) vitamin D coming from the fortified milk (10·5 µg being the total mean daily intake). A similar trend was observed for Fe, in that fortified milk contributed 6·4 mg/d (62 %) of the mean daily intake of 10·4 mg. In terms of Ca intakes, fortified milk consumers obtained 417 mg/d (52 %) from fortified milk, with a total mean daily intake of 795 mg.

Among cows’ milk consumers, dietary supplements were the main contributor to total mean daily vitamin D intakes, contributing 0·4 µg (21 %) of a mean daily intake of 1·9 µg. For cows’ milk consumers, the milk itself did not contribute to the total mean daily Fe intake (5·1 mg), but was the main contributor to Ca intakes, providing 644 mg (65 %) of the mean daily intake of 996 mg.

Nutrient intakes of fortified milk consumers and whole cows’ milk consumers were compared with the EAR and RNI values listed in Table 1. For the fortified milk consumers, 100 % of children had sufficient Fe intake and 99 % had sufficient intake of vitamin D. Among children consuming cows’ milk, the Fe and vitamin D intakes did not meet the EAR for 58 and 92 % of children, respectively (Table 6).

**Table 6.** Proportion (%) of consumers with intake below and above the reference nutrient intake (RNI) or estimated average requirement (EAR)

Nutrient	Fortified milk consumers	Cows’ milk consumers	EAR/RNI
Proportion of consumers with intakes below the EAR/RNI			
Ca	0	0	275 mg/d
Fe	0	58	5·3 mg/d
Zn	0	5	3·8 mg/d
Vitamin A	0	6	300 µg RE/d
Vitamin D	1	92	5·25 µg/d
Iodine	0	0	52·5 µg/d
Proportion of consumers with intakes above the EAR/RNI			
Energy	90	97	3·1 MJ/d (741 kcal/d)
Protein	99	100	14·5 g/d
Na	41	76	775·2 mg/d
Non-milk extrinsic sugars (g/d)	22	16	10 % of total energy
SFA (g/d)	82	100	11 % of food energy

RE, retinol equivalents.

**Fig. 1.**
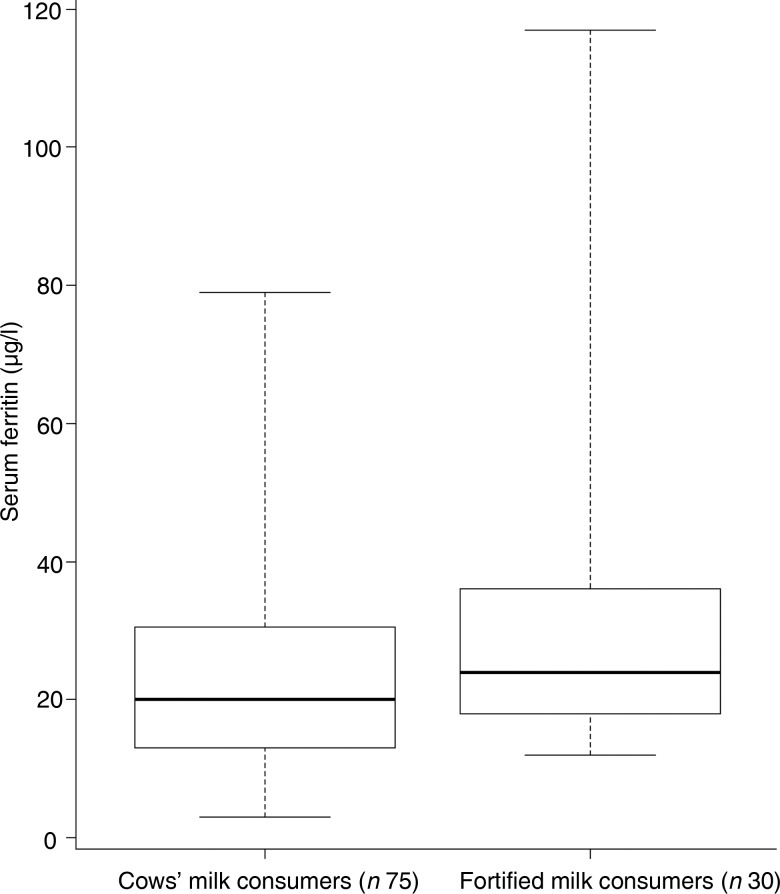
Distribution of serum ferritin (μg/l) concentration in whole cows’ milk consumers and fortified milk consumers. The central lines are medians; the boxes represent interquartile ranges; the whiskers represent ranges.

**Fig. 2.**
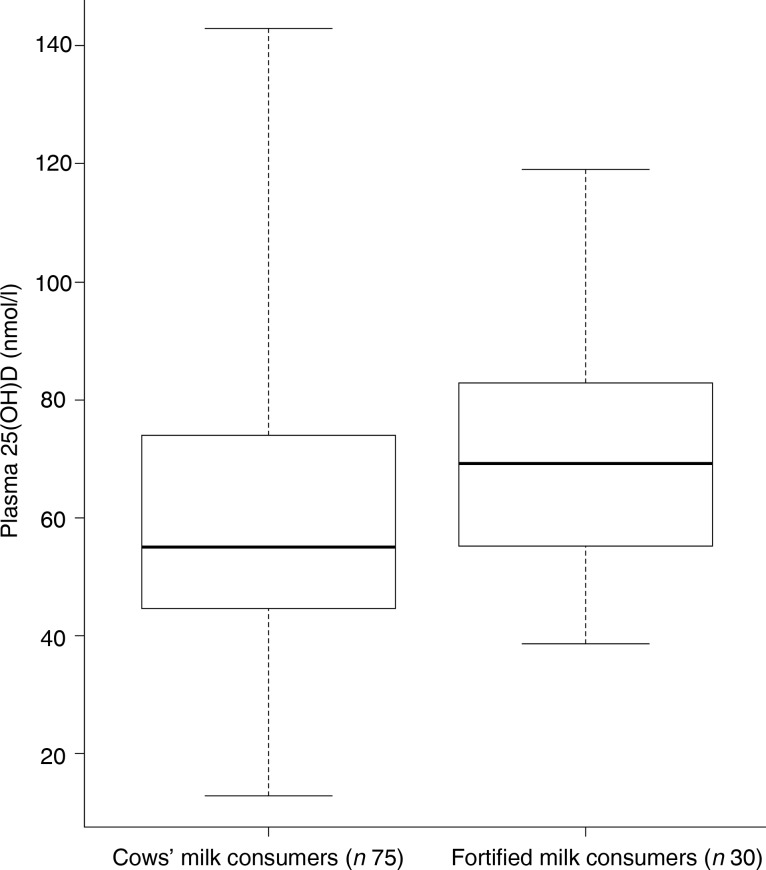
Distribution of plasma 25-hydroxyvitamin D (25(OH)D) (nmol/l) concentration in whole cows’ milk consumers and fortified milk consumers. The central lines are medians; the boxes represent interquartile ranges; the whiskers represent ranges.

The mean Hb concentration did not differ between the two groups (120 g/l for fortified milk and for cows’ milk consumers). Fortified milk consumers had significantly higher mean serum ferritin and plasma 25(OH)D concentrations compared with cows’ milk consumers (*P* < 0·05) (Table 7). The distribution of the data measures for serum ferritin and plasma 25(OH)D are shown in Figs 1 and 2, respectively.

**Table 7.** Comparison of mean serum ferritin and plasma 25-hydroxyvitamin D (25(OH)D) concentrations between whole cows’ milk and fortified milk consumers

	Whole cows’ milk consumers				Fortified milk consumers				
	*n*	Mean	Median	IQR	*n*	Mean	Median	IQR	*P*
Serum ferritin (μg/l)	75	23·7	20	3, 79	30	28·8	22·7	12, 117	< 0·049
Plasma 25(OH)D (nmol/l)	75	60·8	55	12·9, 143	30	73·1	73·3	14·9, 191·8	< 0·014

IQR, interquartile range.

## Discussion

Secondary analysis to identify vulnerable nutrient intakes among young children aged 12–36 months was undertaken in 2013 using a combination of DNSIYC and National Diet and Nutrition Survey data by two of the authors of this study^(^^12^^)^. This provided the stimulus to make a closer examination of the diets of young children aged 12 months and over, to focus on the type of milk that is dominant in their diets, and the effects this has on nutrient intakes, and vitamin D and Fe status. In terms of the daily volume of milk intake that is advised from 12 months of age there is no clear consensus. In terms of meeting Ca requirements the advice is to give at least 300 ml daily^(^^20^^)^, whereas other guidance states ‘around 400 ml’ per d^(^^21^^)^. Guidance to parents in the UK is to give whole (full-fat) cows’ milk from 12 months until at least 24 months rather than reduced-fat milks^(^^22^^)^. The average intake in milk consumers in all DNSIYC participants aged 12–18 months is 434 g/d^(^^2^^)^. Other studies on this topic are based on volumes of milk consumed in a range of 250–500 g/d^(^^23^^,^^24^^)^. Average daily milk intakes for this age group, when milk intake was not restricted, were 445/562 ml for cows’ milk/Fe-fortified milk for consumers in a Swedish study^(^^25^^)^ and >400 g in a New Zealand study^(^^26^^)^. UK manufacturers of fortified milks for this age group suggest 300–400 ml/d^(^^27^^–^^29^^)^.

Milk is commonly consumed by children aged 12–18 months in the UK. Analysis of the DNSIYC data suggests that for those who consume more than 400 g fortified milk/d, nutrient intake is significantly different after 12 months, compared with those who primarily consume cows’ milk. Mean daily intakes of Fe, Zn, vitamin A and vitamin D were significantly higher in the fortified milk group. Mean daily intakes of energy, protein, Ca, iodine, Na and saturated fat were significantly higher in the cows’ milk group. There was no difference in the intake of non-milk extrinsic sugars between the two groups. These intake differences are mainly attributable to the higher concentrations of Fe and vitamin D and a lower protein and saturated fat content in fortified milks compared with whole cows’ milk, and higher concentrations of iodine and Ca in whole cows’ milk. Total daily Ca intake was significantly lower in the fortified milk group, compared with the cows’ milk group; however, all the fortified milk consumers achieved the EAR for Ca. The total saturated fat intake of the fortified milk consumers was significantly lower than that of the cows’ milk consumers. The mean amount of saturated fat contributed by whole cows’ milk was double the amount contributed by the fortified milk. In the formulating of fortified milks, butter fat is typically replaced by vegetable oils, giving a similar total fat to whole cows’ milk, but a more favourable fatty acid profile, i.e. less saturated fat. A limitation of the present analysis is that only children consuming >400 g of each type of milk were included.

Our results are supported by similar studies based on observational data, and also by a number of randomised controlled trials. A small study using data from the Irish National Pre-School Nutrition Survey (2010–2011) compared the nutrient intakes of children consuming at least 100 g of fortified milk within a total milk intake of 300 g/d or more, with those consuming only cows’ milk (>300 g/d). Intakes of protein, saturated fat and vitamin B_12_ were lower and intakes of carbohydrate, Fe, Zn, and vitamins C and D were higher in consumers of fortified milk^(^^30^^)^. The authors attributed these results mainly to the differences in composition between fortified milk and cows’ milk and concluded that fortified milk consumption reduced the risk of inadequacies of Fe and vitamin D.

A further cross-sectional study from France reported that children aged 1–2 years who consumed >250 g/d of fortified milk had a lower risk of insufficiency (measured as proportion below EAR) of Fe and vitamin D (and also vitamin C and α-linolenic acid), compared with those consuming >250 g/d of cows’ milk^(^^23^^)^. Intake of other foods could not account for the differences. A risk of inadequacy remained for vitamin D in the group consuming fortified milk, but this may be explained by the exclusion of supplements and a higher vitamin D recommended daily intake in France (10 µg/d, compared with the UK requirement of 7 µg/d).

In addition to these observational studies, randomised controlled trials provide evidence of the effectiveness of Fe-fortified formula on maintaining Fe status. However, few studies have been conducted in children older than 12 months of age. One Spanish study assigned thirty-three healthy 1- to 3-year-olds to receive 500 g/d of Fe-fortified milk or 500 g/d of unmodified cows’ milk. After 4 months, the fortified milk group showed significantly higher serum ferritin and lower serum transferrin concentrations than the cows’ milk group^(^^24^^)^. Two studies have investigated the effect of Fe-fortified cows’ milk in children aged around 12 months with adequate Fe status. Fortified milks (1·5 mg Fe/100 g) were consumed for 5 to 6 months in Sweden and New Zealand. In the Swedish study^(^^25^^)^, Fe intakes in the unfortified group at 18 months (mean 5·84 mg/d) were low in relation to Nordic Nutrition Recommendations (8 mg/d for 1- to 3-year-olds), while the intakes in the Fe-fortified group (10·87 mg/d) were acceptable. There were no significant differences in blood Hb, serum ferritin, serum Fe and transferrin Fe saturation in the participants consuming the fortified diet during the study period and serum ferritin just failed to reach significance. The New Zealand-based study was a 20-week trial to explore the efficacy of an increased intake of either red meat, with an advised consumption of two meat meals per d (contributing 2·6 mg/d Fe), or Fe-fortified milk formula (Fe concentration 1·5 mg/100 g), on improving Fe status in non-anaemic young children, compared with controls consuming non-fortified powdered milk. Mean consumption of fortified milk was 400 g/d contributing on average 6 mg/d of Fe. Serum ferritin increased by 44 % in the fortified milk group, did not change significantly in the red meat group, and tended to decrease in the control group. By 20 weeks, in comparison with the control group, serum ferritin was significantly higher in the fortified milk group^(^^26^^)^.

A recent review of strategies (mainly in low- to middle-income countries) to prevent Fe-deficiency anaemia in older infants identifies micronutrient sprinkles, Fe-fortified milk, Fe supplementation and food-based strategies as possible solutions, and concludes that fortified milk is an effective strategy^(^^31^^)^. Conversely, high consumption of cows’ milk (more than 500 ml/d) has been identified as a risk for Fe deficiency^(^^32^^)^.

With regards to vitamin D intakes and status, evidence also suggests that fortified milk can reduce the risk of insufficiency. Although the UK has a supplementation policy for infants and young children, supplement uptake is often poor: data from the DNSIYC highlighted that only 9 % of children aged 12–18 months were given a vitamin D supplement during the 4-d recording period^(^^2^^)^. Vitamin D drops are not regarded as necessary if the child is consuming more than 500 g of fortified formula. However only 32 % of non-breastfed children were consuming fortified milk at 12–18 months, with a mean intake of less than 400 g/d^(^^2^^)^. Thus, the problem of low dietary vitamin D intakes persists as supplements are not widely consumed, and few other dietary sources commonly consumed by young children currently provide significant amounts of vitamin D. Vitamin D deficiency among young children may be a real cause for concern, with the Scientific Advisory Committee on Nutrition (SACN) currently drafting new guidelines for consultation (recommending 10 µg/d compared with the current recommendation of 7 µg/d for this age group)^(^^33^^)^. Plasma 25(OH)D concentrations were significantly higher among the children aged 12–18 months consuming >400 g/d of fortified milk in the DNSIYC, although there was only a percentage of this subgroup from whom blood samples were available for analysis (Table 2). Vitamin D status is generally low in winter and spring and higher in summer and autumn. However, the blood sampling conducted in the DNSIYC was carried out in two waves: February to May 2011, and April to August 2011, and therefore cannot be considered as giving a year-round average vitamin D measure^(^^2^^)^.

Guidance to parents in the UK is to give whole (full-fat) cows’ milk from 12 months until at least 24 months rather than reduced-fat milks^(^^22^^)^ and that young children from 1 year of age should be offered about three portions of milk and dairy foods per d^(^^34^^)^. In terms of protein intakes which are higher than necessary, it may be preferable to recommend a reduced-protein milk for this age group given the emerging evidence that protein in excess of requirements may encourage rapid early growth and contribute to later adiposity. Since milk is a valuable, economical and nutritious food, making an important contribution to key nutrients in the second year of life, a reduced-protein, Fe and vitamin D-fortified formula designed for young children may contribute to a more favourable dietary intake pattern regarding energy, protein and micronutrients, and a better status of Fe and vitamin D.

A limitation of this study was that only intakes of certain nutrients and Fe and vitamin D status were examined. We did not attempt to examine socio-economic factors related to the subgroups. It is possible that a decision to feed cows’ milk or fortified milk is influenced by income and education factors of families or other factors. Fortified milk is not the only means of ensuring adequate dietary Fe and vitamin D intakes in young children aged 12–18 months. However, the inadequate intakes of essential nutrients among consumers of cows’ milk suggest that other complementary dietary recommendations (such as regular consumption of red meat/fish/eggs, supplements or fortified foods) are not being adhered to, resulting in potential deficiency of these micronutrients among this age group.

### Conclusion

Most young children in the UK consume whole cows’ milk, fortified milk or both. This study documents significant differences in intakes of vitamin D, vitamin A, Zn and Fe between young children consuming more than 400 g/d of fortified milk *v*. cows’ milk. Fortified milk allowed almost 100 % of young children to meet their requirements of vitamin D and Fe, and in the subset measured, it improved vitamin D and Fe status. Finally, fortified milk consumers met the requirement for Ca, as did all milk consumers, but without increasing intakes of energy, protein and saturated fat, which are already high in this population. These results suggest that fortified milks can play a significant role in optimising the diet quality of young children in their second year of life.
